# Effective coverage of newborn postnatal care in Ethiopia: Measuring inequality and spatial distribution of quality-adjusted coverage

**DOI:** 10.1371/journal.pone.0293520

**Published:** 2023-10-26

**Authors:** Aster Ferede Gebremedhin, Angela Dawson, Andrew Hayen

**Affiliations:** 1 Department of Public Health, College of Health Sciences, Debre Markos University, Debre Markos, Ethiopia; 2 School of Public Health, University of Technology Sydney, Sydney, Australia; National Research Centre, EGYPT

## Abstract

Neonatal health is a significant global public health concern, and the first two days of life are crucial for newborn survival. Most studies on newborn postnatal care have focused on crude coverage measures, which limit the evaluation of care quality. However, evidence suggests a shift towards emphasising effective coverage, which incorporates the quality of care when measuring intervention coverage. This research aimed to assess the effective coverage of newborn postnatal care in Ethiopia while also examining its inequalities and spatial distribution. The study used secondary data from the 2016 Ethiopian Demographic and Health Survey, which was a cross-sectional community-based study. A total weighted sample of 4169 women was used for analyses. We calculated crude coverage, which is the proportion who received a postnatal check within 48 hours of birth and quality-adjusted coverage (effective coverage), which is the proportion who received a postnatal check within 48 hours of birth and reported receipt of 6 or more contents of care provided by health care providers. Concentration index and concentration curves were used to estimate the socioeconomic-related inequalities in quality-adjusted newborn postnatal care. The spatial statistic was analysed by using Arc-GIS. The crude coverage of newborn postnatal care was found to be 13.2%, while the effective coverage was 9%. High-quality postnatal care was disproportionately concentrated among the rich. A spatial variation was found in quality-adjusted coverage of newborn postnatal care across regions. The findings suggest that there is a significant gap in the coverage and quality of postnatal care for newborns across regions in Ethiopia. The low rates of coverage and effective coverage, combined with the concentration of high-quality care among the rich and the spatial variation across regions, highlight the need for targeted interventions and policies to address the inequalities in access to high-quality postnatal care for newborns.

## Introduction

In the global health system, neonatal health is a critical issue illustrating progress and challenges on both the national and global levels. Reducing neonatal mortality is a critical component of the third sustainable development goal to end preventable deaths in children [1]. Postnatal care delivers essential and evidence-based interventions that reduce morbidity and mortality rates among mothers and their children [2]. According to UNICEF’s 2022 report, 69% of mothers and only 66% of newborns received a postnatal health check worldwide. Access to and uptake of newborn postnatal care has improved but remains limited in low- and middle-income countries. In sub-Saharan Africa, the magnitude of newborn postnatal care is highly variable and very low, for instance, 20% in Namibia, 21% in Angola, 13% in Ethiopia, 36% in Kenya, 38% in Nigeria, and 56% in Uganda [3].

Since 1990, considerable progress has been made globally in improving child survival. The number of neonatal deaths has declined from 5 million in 1990 to 2.4 million in 2020. However, the reduction in neonatal mortality has been slower compared to post-neonatal under-5 mortality. Neonatal deaths vary widely across regions globally. Most occur in low-income countries, where newborns are almost ten times more likely to die than in high-income countries. In 2020, Sub-Saharan Africa had the highest neonatal mortality rate of 27 deaths per 1000 live births, followed by central and southern Asia with 23 deaths per 1000 live births [4]. The Ethiopian Demographic and Health Survey (EDHS) 2016 report revealed that the neonatal mortality rate of the country is 29 deaths per 1000 live births [5].

Neonatal deaths constitute an increasing share of global under-five deaths, indicating a generalised need to strengthen neonatal programs [6]. Most neonatal deaths occur in the first two days of life-from conditions and diseases associated with lack of quality care- which makes the postnatal period critical to improving child survival. Interventions targeted at the first week of life are likely to have the greatest impact on neonatal survival. It is essential that newborns receive high-quality postnatal care [7].

Crude coverage measures, often used in MCH studies, are the tip of the iceberg as they focus only on contact between women or children and health care providers. They provide no indication of the extent of adherence to care standards, the way services are provided, and the quality of care delivered. In contrast, effective coverage combines utilisation of health services with the quality of care received, providing a proxy measure of potential health gains from using the services [8]. Although assessing quality is the most challenging aspect of estimating effective coverage, integrating content of care into crude coverage measures has been proposed as one approach to determining quality-adjusted coverage [9]. While data collected through DHS do not contain the full recommended list of services or important clinical actions, they provide an indication of care provided at service delivery sites (content of care), at a basic level, as a proxy for quality of care. Hence, DHS data provide information that can assist in the assessment of whether the minimum expected services are received by women or children [10]. In this study, we assessed the crude and quality-adjusted coverages of newborn postnatal care in Ethiopia using a nationally representative survey. We then examined the inequalities in and the spatial distribution of quality-adjusted coverage of newborn postnatal care.

## Methods

This is a cross-sectional study conducted in Ethiopia using the 2016 EDHS, which is the fourth survey in the country.

### Data source

We conducted a secondary analysis of the 2016 EDHS data. DHSs are nationally representative household surveys that provide data for a wide range of monitoring and impact evaluation indicators in the areas of population, health, and nutrition. So far, four full-scale/major DHSs (2000, 2005, 2011, and 2016) have been conducted in Ethiopia.

Ethiopia is a landlocked country located in the Horn of Africa and bordered by Eritrea in the north, Djibouti in the northeast, Sudan in the west, Somalia in the east and northeast, and Kenya in the south. Administratively, Ethiopia is divided into nine regions (Tigray, Afar, Amhara, Benishangul-Gumuz, Gambela, Harari, Oromia, Somali, and Southern Nations Nationalities and People’s (SNNP)) and two city administrations (Addis Ababa and Dire Dawa). The EDHS 2016 was a cross-sectional household study implemented by the Ethiopian Central Statistical Agency as part of the worldwide MEASURE DHS project. The survey’s sampling frame used the Ethiopia Population and Housing Census from 2007, and the survey included all nine regions and two city administrations in Ethiopia, with a two-stage stratified cluster sampling technique. Stratifications were done based on separating each region into urban and rural areas. Probability sampling was used to select 645 enumeration areas (EA), including 202 urban and 443 rural areas, in the first stage. The second stage involved selecting 28 households systematically in each EA, leading to complete interviews at 16,650 households. The survey interviewed a total of 15,683 reproductive-aged women (15–49) and 12,688 men aged 15–59.

The EDHS 2016 survey utilised four different types of questionnaires to obtain primary data, including the household questionnaire, woman’s questionnaire, man’s questionnaire, biomarker questionnaire, and health facility questionnaire. These questionnaires, which were adapted to address population and health issues relevant to Ethiopia, followed the DHS Program’s standard Demographic and Health Survey questionnaires. The Woman’s Questionnaire was used to collect information from all eligible women aged 15–49. To ensure appropriate quality control, the EDHS survey utilised pre-testing, staff training, and close supervision of data collectors during fieldwork. The data collection spanned a period of 5.5 months, commencing on January 18, 2016, and concluding on June 27, 2016. To conduct the fieldwork, a total of 33 field teams were assembled, each comprising a team supervisor, a field editor, three female interviewers, one male interviewer, two biomarker technicians, and one driver. To ensure the quality and integrity of the data, an additional 28 quality controllers were deployed throughout the data collection process. Their role was to provide support and oversee the fieldwork activities. During the data collection process, clear explanations of the questions were provided to respondents to ensure their understanding. For the seamless transfer of electronic data files, a secure system called Internet File Streaming System was utilised, enabling regular and secure transmission of the files to the central office of the Central Statistical Agency (CSA) in Addis Ababa. The coordination and supervision of the fieldwork operations were jointly carried out by the CSA, Federal Ministry of Health, Ethiopian Public Health Institute, and experts from the DHS Program [5]. A detailed methodology is available in the EDHS report, which can be accessed at https://dhsprogram.com/.

### Study population

We utilised the Individual Women Record (IR) file from the 2016 EDHS dataset, encompassing data on 15,683 women. From this extensive dataset, we extracted data pertaining to all women of reproductive age (15–49) who had given birth in the two-year preceding the survey. Although DHS indicators are calculated based on a five-year reference period, the recommended indicators for postnatal care are based on births in the two years preceding the survey [11]. We, therefore, included only women who met this criterion. We further refined our data by excluding 131 observations with missing values, resulting in a final weighted sample of 4,169 women.

### Study variables

Crude (contact) coverage was defined as the proportion of newborns born alive in the previous two years whose mothers reported that they had a post-natal check within 48 hours of birth. Quality of care was measured based on the content of care that newborns received during their contact with health care providers. We selected 11 newborn intervention items: a woman reported her baby receiving the following services: BCG vaccination, the first polio vaccine, not given prelacteal feed, early initiation of breastfeeding, temperature measured, counselling on danger signs, cord examined, counselling on breastfeeding, observation of breastfeeding, newborn weighted, and skin to skin contact. The response to each question/item was coded as ‘1’ if a particular service was received and as ‘0’ otherwise. An index of newborn postnatal care quality was created by adding all “yes” responses for each woman. Based on previous evidence [12], we classified the quality of care as “high quality” if the women scored above 5 of the 11 interventions corresponding to the 75^th^ percentile. To ascertain the internal consistency and validity of the 11 items in relation to the quality construct, Cronbach’s reliability coefficient was computed. We found that Cronbach’s α reliability coefficient was 0.77 for the full set of coverage indicators. The outcome variable of this study was quality-adjusted newborn postnatal care coverage (effective coverage). It was defined as the percent of newborns who had a postnatal check within 48 hours of birth and for whom all six or more contents of care were met (high-quality care).

### Analytical approach

The cleaned and recoded data were analysed using STATA 14. Frequencies and percentages were used to summarise the characteristics of variables. Data were presented using tables and graphs. We calculated the proportions of crude coverage, receipt of high-quality care, and quality-adjusted coverage of newborn postnatal care. The 2016 EDHS survey aimed to provide representative data at both national and subnational levels. However, since population distribution varies across regions, proportional allocation alone may not yield sufficient sample sizes for reliable estimates in smaller regions. To address this, regions with smaller populations were oversampled, while those with larger populations were under sampled. Nevertheless, this intentional oversampling and under sampling may lead to biased estimates. To restore representativeness, DHS applied sampling weights to the data, ensuring it reflected the actual distribution of Ethiopia’s population. Sample weights were calculated by sampling experts and included in each DHS recode file. We used the sampling weight v005/1,000,000 and the STATA “*SVY*” command whenever we run estimates. A detailed explanation of the weighting procedure can be found in the EDHS final report [5].

We used the concentration index to estimate the socioeconomic-related inequalities in quality-adjusted newborn postnatal care. Concentration curves were used to display the degree of inequality by plotting the cumulative percentage of quality-adjusted coverage on the Y-axis against the cumulative percentage of the population ranked by household socioeconomic status (starting from the poorest and ending with the richest) on the X-axis [13]. If the health outcome is perfectly equally distributed across the population, the concentration curve will be a 45-degree (diagonal) line, known as the line of equality. A curve that is farther from the line of equality indicates a greater degree of health inequality. A concentration curve that falls below the line of inequality indicates that quality-adjusted coverage is high among the rich (pro-rich); a curve that falls above the line of inequality indicates that coverage is high among the poor (pro-poor). The concentration index, which ranges from -1 to +1, is twice the area between the concentration curve and the line of inequality.The concentration index value will be negative when the outcome is disproportionately concentrated among the poorest quintiles. In contrast, the concentration index will be positive when the outcome is more concentrated in wealthier quintiles. If there is no socioeconomic inequality, the concentration index will be zero [13]. The user-written STATA commands *conindex* [14] and *Lorenz* [15] were used to measure concentration index and produce concentration curve, respectively. The spatial statistic was analysed by Arc-GIS version 10.3 software. Hot Spot Analysis of the z-scores and significant p-values (Getis-Ord Gi* statistic) identifies locations with hot or cold spot values concentrated in space. In this study, hot spot areas with high clusters of high-quality newborn postnatal care and cold spot areas with low-level clusters were identified.

## Ethics

This study utilised a publicly available data source, and therefore, ethical approval was not required, and informed written or verbal consent was not applicable. No participants were recruited, and no access to medical records was sought. The authorisation for using the data was granted by the DHS program. According to the EDHS 2016 report, all participant data was anonymised during data collection [5].

## Results

### Sociodemographic characteristics

The study included a total weighted sample of 4169 women. Nearly half 2127 (51%) of the respondents were 25–34 years old. The majority of the respondents (96%) were married or living with their partners. It was found that 3,674 (88%) of the women were rural residents. Furthermore, 60% and 58% of the women had no formal education and were home makers, respectively. The highest number of participants 1841(44%) were from Oromia region (**Table 1**).

**Table 1:** Background characteristics of the study population, Ethiopia Demographic and Health Survey 2016.

Variable	*n = 4169* (Weighted)	Percentage
**Women’s age**		
15–24	1,219.1	29.2
25–34	2,126.6	51.0
35–49	823.1	19.7
**Marital status**		
Not married /not in union	187.1	4.5
Married/living with partner	3,981.8	95.5
**Residence**		
Urban	494.7	11.9
Rural	3,674.2	88.1
**Region**		
Tigray	306.9	7.4
Afar	41.0	1.0
Amhara	768.9	18.4
Oromia	1,840.9	44.2
Somali	170.2	4.1
Benishangul	44.1	1.1
SNNPR	852.4	20.5
Gambela	9.7	0.2
Harari	9.9	0.2
Addis Ababa	107.3	2.6
Dire Dawa	17.6	0.4
**Mother’s education**		
No education	2,514.5	60.3
Primary	1,284.2	30.8
Secondary and higher	370.1	8.9
**Mother’s occupation**		
Not working	2,426.8	58.2
Professional work	671.6	16.1
Non-professional work	1,070.5	25.7
**Income levels**		
Q1 (Lowest 20%)	971.8	23.3
Q2	927.0	22.2
Q3	861.1	20.7
Q4	766.4	18.4
Q5 (Highest 20%)	642.6	15.4

### Crude and effective coverage of newborn postnatal care

The crude coverage of newborn postnatal care, as measured by the proportion of women whose newborns received a postnatal check within two days of birth, was 13.2%. The effective coverage of newborn postnatal care was 9% (Table 2).

**Table 2:** Crude and effective coverage of newborn postnatal care and 95% confidence intervals (CI), Ethiopia Demographic and Health Survey 2016.

Variables	Yes (95% CI)	No (95% CI)
Crude coverage (received a postnatal check within 48 hours of birth)	13.19(11.24–15.41)	86.81(84.59–88.79)
Effective coverage (received a postnatal check within 48 hours of birth and reported receipt of six or more contents)	9.05(7.53–10.83)	90.95(89.17–92.47)

**Fig 1:**
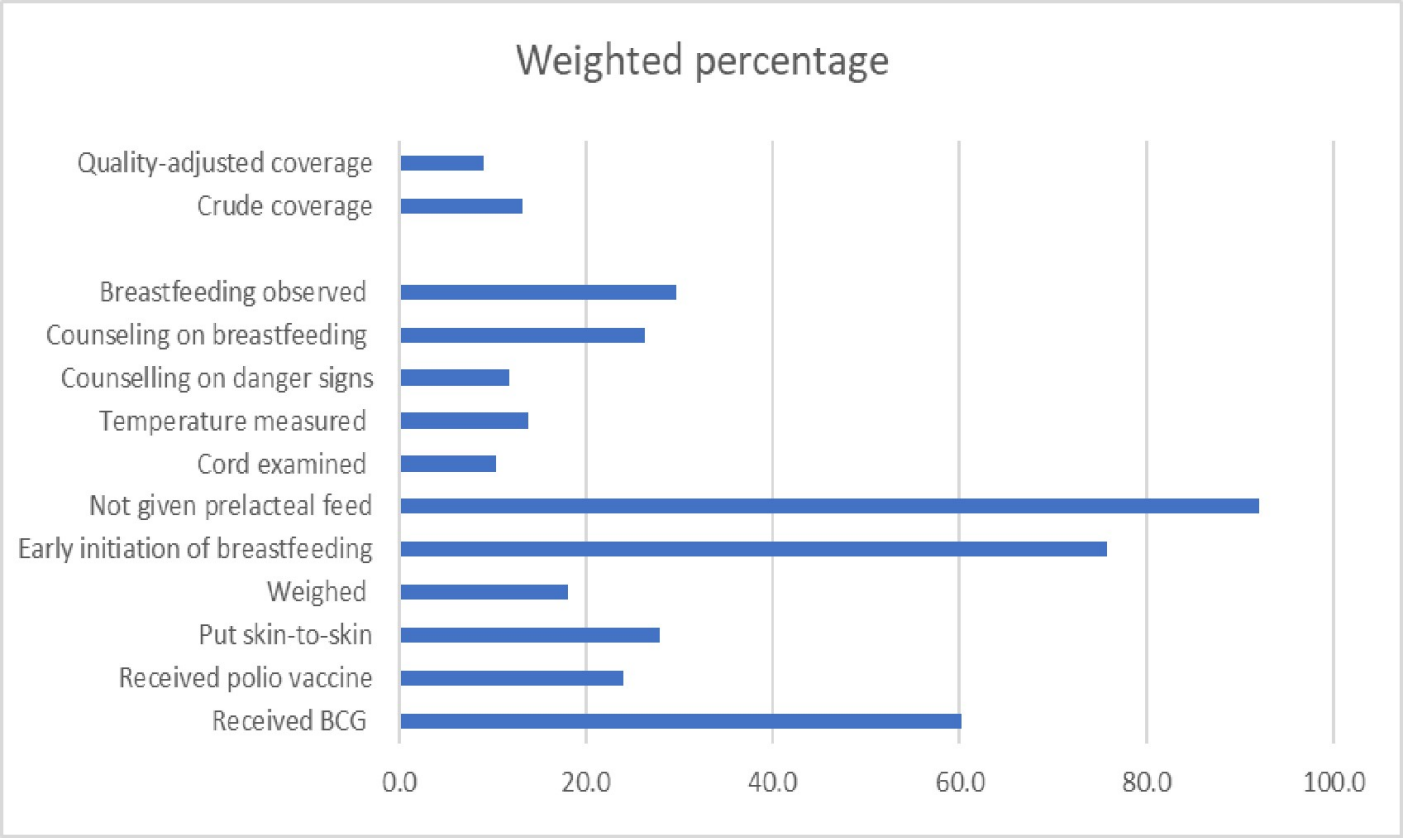
Contents, crude coverage and quality-adjusted coverage of newborn postnatal care, Ethiopia Demographic Health Surveys 2016.

**Fig 1** shows the contents of newborn postnatal care. Among the women who gave birth in the two years preceding the survey, the proportion of women receiving specific interventions during newborn postnatal care was 60%, 24%, 28%, and 18% for BCG vaccination, polio vaccination, skin-to-skin contact with the mother, and weight measurement, respectively. Three-fourths of the women, 3,159 (76%) initiated breastfeeding within one hour of birth. More than 90% of the newborns were not given prelacteal feeds. The proportion of women who reported that their newborn’s cord was examined, temperature was measured, and breastfeeding was observed was 10%, 14%, and 30% respectively. About twelve percent of the women reported receiving counselling on danger signs, while 26% reported counselling on breastfeeding.

Overall, out of the 11 newborn intervention items, 22% of the newborns received six or more interventions. Effective coverage of newborn postnatal care, which denotes high-quality or quality-adjusted newborn postnatal care, was measured by the proportion of women whose newborns received a postnatal check within two days of birth and having received at least six newborn intervention items. It was found that the effective coverage of newborn postnatal care was 9.1% (n = 377). (See **Fig 1**).

### Socio-economic inequality in quality-adjusted newborn postnatal care

**Fig 2:**
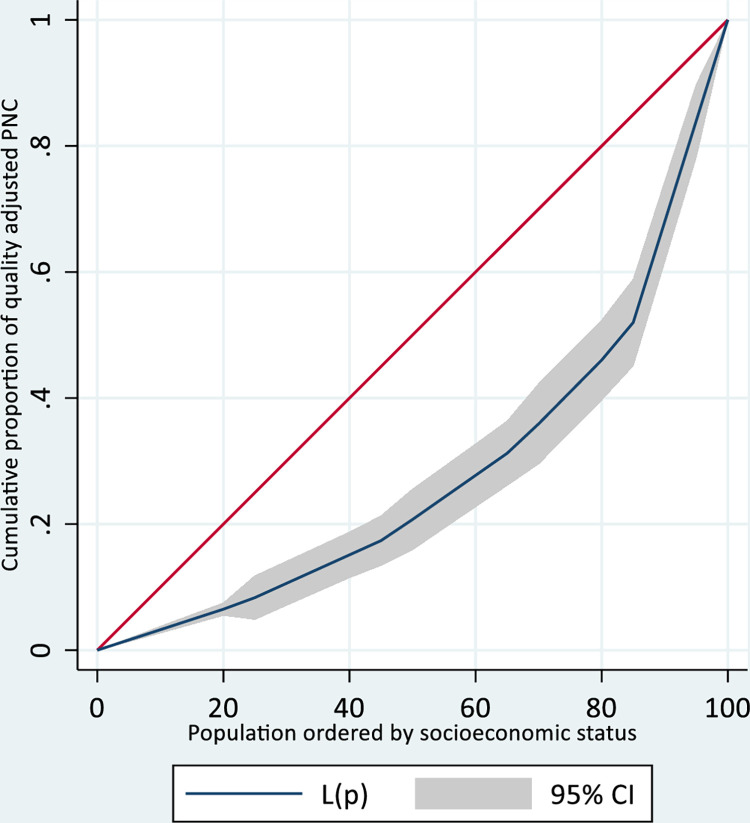
Concentration curve of quality-adjusted newborn postnatal care, Ethiopia Demographic Health Surveys 2016.

**Fig 2** indicates that quality-adjusted postnatal care for newborns (high-quality postnatal contact) was disproportionately concentrated among the rich (pro-rich) as the concentration curve lies below the line of equality. The result of the concentration index also showed a pro-rich distribution (concentration index = 0.439, standard error = 0.0458) (**Fig 2**).

### Spatial distribution of quality-adjusted coverage of newborn postnatal care

**Fig 3:**
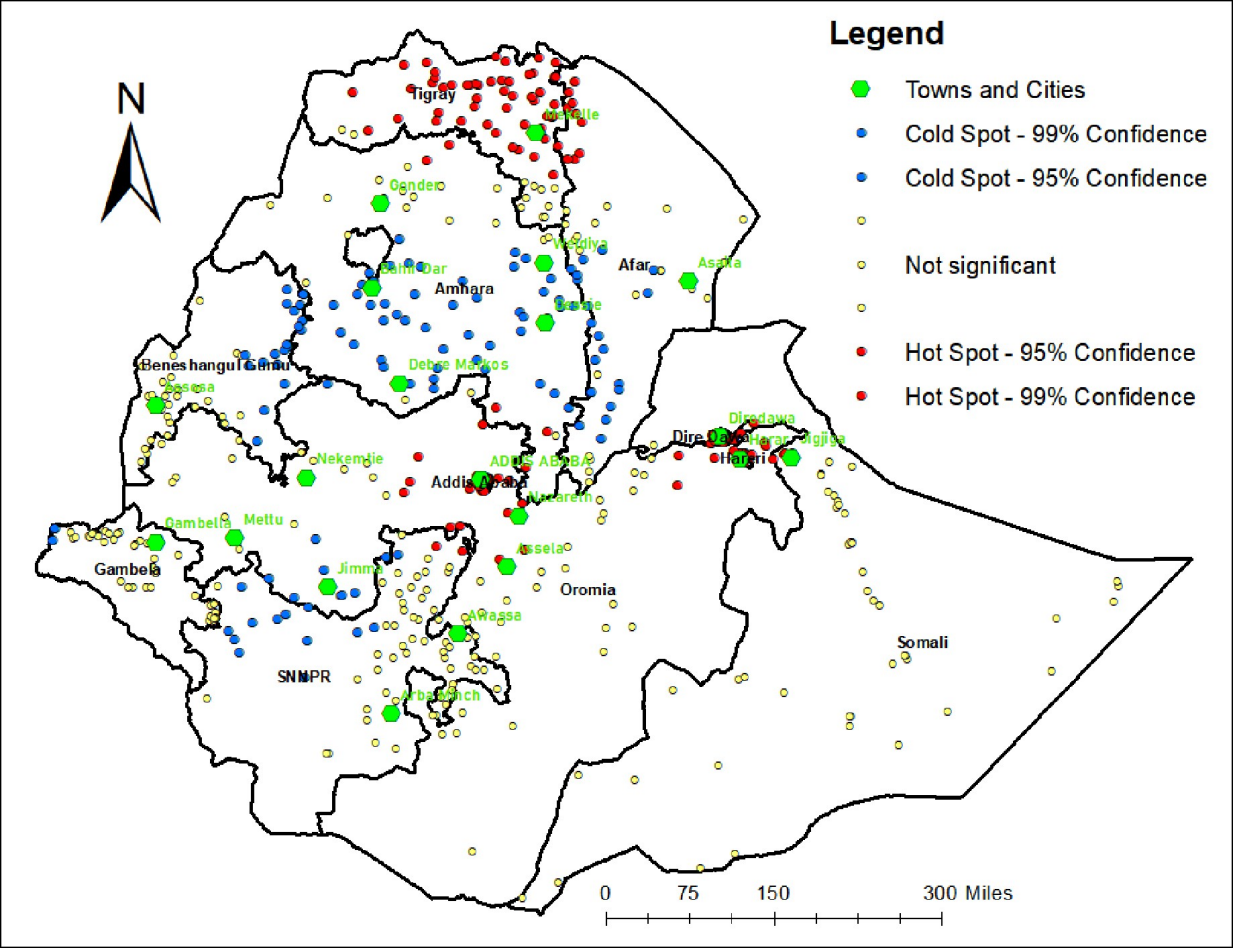
Hot spot analysis of quality-adjusted coverage of newborn postnatal care, Ethiopia Demographic and Health Survey, 2016.

**Fig 3** shows that spatial variation was found in quality-adjusted coverage of newborn postnatal care at regional levels. A total of 622 clusters were considered for the spatial analysis of quality-adjusted coverage of newborn postnatal care. Each point on the map represents one enumeration area with the proportion of quality-adjusted coverage of newborn postnatal care in each cluster. The red circles indicate statistically significant spatial clusters with high-quality newborn postnatal care (hot spot areas), while the blue circles represent statistically significant spatial clusters with low-quality newborn postnatal care (cold spot areas). It is worth noting that the individual data points do not reveal the specific locations of respondents. Instead, the coloured circles represent aggregated spatial clusters. Significant hot spot areas (red colours) were detected in Tigray, Addis Ababa, Dire Dawa, Harari, central parts of Oromia, and SNNPR (northern parts). In contrast, significant cold spot areas (blue colours) were detected in central and southern parts of Amhara, central and southwestern Afar, eastern parts of Benishangul Gumuz, western parts of Gambela, northern parts of SNNPR, and western parts of Oromia (**Fig 3**).

## Discussion

We found that the coverage of newborn postnatal care was low in Ethiopia. In addition, high-quality contact for newborns was lower than the crude coverage estimate. We also found that socioeconomic inequalities exist in the quality-adjusted coverage of newborn postnatal care in favour of the rich (pro-rich). Furthermore, this study provided a visually powerful analysis of the spatial variation in quality-adjusted coverage of newborn postnatal care in Ethiopia.

Evidence shows measuring contact with a health service alone is not sufficient to assess the true population receipt of health services. Crude coverage indicators-often used in coverage metrics- may overestimate the health gains of interventions as they do not reflect the quality of the care delivered. As a result, adjusting crude coverage measures for quality of care has become a key area of interest in coverage metrics [16]. We applied the concept of effective coverage metrics by incorporating contents of care (quality dimension) into the coverage estimate of newborn postnatal care. Our findings highlight that quality lagged coverage and there was a quality-coverage gap for newborn postnatal care. This gap indicates that along with the contact coverage of newborn postnatal care, there is a need to focus on the quality of care being delivered. Low quality of care undermines the effective delivery of essential health services [17]. Our finding was consistent with other studies that noted a decline in the coverage of maternal and child health interventions on adding quality of care to their metrics [18, 19]. This means that women/children who sought care in health care facilities did not necessarily receive the standard quality of care they needed.

The quality-adjusted coverage estimate of the present study was higher than the coverage reported in the study by Marchant et al. 2015 [20]. This might be due to the difference in the study setting and sample selection. The study by Marchant et al., 2015 considered four regions of Ethiopia, Gombe state (Nigeria), and a state in India, and the study participants represented a program of work in each setting at baseline [20]. Similarly, our quality-adjusted coverage estimate is higher than the figure reported in the Côte d’Ivoire study [21]. The disparity could be attributed to the difference in methods used such as quality measurement. Munos et al. linked health provider assessment and care-seeking data and used process and structure dimensions to measure the quality of newborn care [21]. Recent studies focus on effective coverage evaluation that depends on linking data on need and service utilization from population-based surveys, with data on service quality from health facility surveys such as the service provision assessment. In such cases, either the summary of the facility’s capacity to provide high-quality care is linked with individual data, or individuals are linked to their reported source of care. Such methods overlook variation in the quality of care both within and between health facilities [22].

In contrast, our estimate is lower than Mexico’s study, which revealed a quality-adjusted coverage estimate of 74% [23]. The variation in the estimates might be attributable to differences in how crude coverage and quality were defined and measured. The Mexican study defined newborn care as the proportion of live newborns who were delivered in Mexican Institute of Social Security facilities in relation to live births in the previous year. This study used the proportion of live births reaching 28 days without death due to respiratory infection, nosocomial infection, or sepsis as a quality indicator that corresponds to an outcome dimension of quality [23]. Across maternal and child health studies, the approaches and strategies used to evaluate the quality component of effective coverage are different. The quality dimensions considered, or the selection of indicators determine the effective coverage estimate. However, quality is a multidimensional, complex construct with few standardized and validated measures [9].

The estimated concentration index and concentration curve suggest considerable socioeconomic inequality in the quality-adjusted coverage of newborn postnatal care and indicate that the use of high-quality newborn postnatal care was relatively higher among households with high socioeconomic status. This is in line with previous survey-based evidence from low and middle-income countries [24]. Inequalities in MCH coverage indicators favouring the rich are common. Often, women with high socioeconomic status have better access to quality care and can afford the medical, non-medical, and opportunity costs of newborn care. Such women are also considered to be more empowered and autonomous than their poorer counterparts [25, 26]. Assessing socioeconomic inequality using contact coverage measures may not reveal disparity adequately. Evidence shows widening inequalities in the quality of MCH care may lead to widening socioeconomic inequalities in effective coverage. Hence, improving quality may contribute to attaining equitable high-quality health care [27].

The economic, social, political, and infrastructural dynamics of a country or region fundamentally shape the outcomes in healthcare [28]. In addition to highlighting socioeconomic inequalities, which indicate that certain population groups lag behind in accessing high-quality newborn care, the findings of this study reveal distinct regional variations in the distribution of high-quality newborn postnatal care. Most of the areas identified as hotspots in the spatial statistics are relatively urban, and these areas generally have a higher human development index compared to the cold spot areas [29, 30]. Previous evidence from spatial and multivariable analyses revealed that these regions had high postnatal care coverage [31, 32]. Urban areas in Ethiopia have better access to healthcare facilities, infrastructures and resources, including skilled healthcare professionals, compared to rural areas. For example, Addis Ababa, Dire Dawa and Harari city administrations, with urban population proportions of 100%, 63% and 56%, respectively, demonstrate the highest health workforce to population ratio. Conversely, regions like Amhara and Afar, with less than 15% urban population, have a lower ratio, indicating a disadvantage in accessing health professionals compared to their urban counterparts [33, 34]. Women residing in urban areas and those with higher incomes typically enjoy better proximity to healthcare services, enabling timely interventions both prenatally and post-delivery, which improve neonatal outcomes [35]. Additionally, these women often have increased exposure to health information and access to educational resources, and support groups, promoting awareness and enabling informed decisions about prenatal and neonatal health, preventive measures, and appropriate care for both mother and newborn. Furthermore, such women are more likely to receive regular and comprehensive antenatal care. Prior research has indicated that antenatal care attendance is strongly associated with higher quality of maternal and neonatal postnatal care, as it provides an increased opportunity for mothers to become more informed about potential complications and receive greater encouragement to seek high-quality postnatal care [36]. It is worth mentioning that cold-spot clusters exhibited in the relatively rural, remote or border areas of Ethiopia suggest that women from such areas might have limited access to MCH and other public services [37].

Research on the effective coverage of newborn postnatal care is limited [9]. Most of the studies conducted in Ethiopia concerning newborn postnatal care focused on crude coverage [38–41]. Peven et al., 2021 assessed the gap between postnatal contact coverage and content coverage using survey data from low and lower-middle-income countries [42]. Furthermore, Marchant et al. 2015 estimated the coverage of high-quality contacts in three study sites including Ethiopia [20]. However, the populations sampled in this study represented only a program of work at baseline. None of the above-mentioned studies examined the inequality and spatial distribution of quality-adjusted newborn postnatal care in Ethiopia. This study is unique in that it employed spatial analysis to indicate hotspot areas of high-quality newborn postnatal care. Furthermore, the results presented are in line with SDG 17.18, which calls for the disaggregation of health and related indicators based on different dimensions of inequality, including socioeconomic status [43].

This study has some limitations. The DHS is based on self-reporting, and recall bias could affect the reported measures of the different services received by women and children. Additionally, due to limited information collected by the DHS, our measurement of the quality of care did not adjust for facility readiness and service provision and did not consider the multiple domains of quality of care that may further reduce the effective coverage estimate. Thus, the quality coverage estimates only reflect the minimum conditions required for judging the quality of care and may lead to overestimation. The other key limitation of our study is the utilisation of the 2016 EDHS dataset, which was conducted seven years before the current date of August 2023. While this dataset provides invaluable insights into past trends and patterns, it may not entirely capture the present dynamics and transformations in health coverage and inequality. As time progresses, the context surrounding health interventions and policies is subject to evolution, potentially rendering some aspects of the dataset less reflective of the current landscape. However, it is imperative to acknowledge that despite this limitation, the 2016 EDHS remains the most recent and comprehensive large-scale survey available for our research.

By examining the elements that constitute a comprehensive approach to newborn postnatal care, our study defined ’high-quality’ newborn postnatal care based on six or more care measures, as identified through a literature review. However, we recognise the potential benefits of conducting further investigation in this area. Future research efforts should focus on identifying which specific components of the neonatal checkup have the most significant impact on reducing newborn deaths.

## Conclusions

This study shows that the effective coverage of newborn postnatal care in Ethiopia was low, and there was a quality-coverage gap. The study also revealed that high-quality newborn postnatal care is disproportionately concentrated among the rich. Hot spot areas of high-quality newborn postnatal care were detected in Tigray, Addis Ababa, central parts of Oromia, SNNPR, Dire Dawa, and Harari, while the cold spot areas were detected in Amhara, central and southwestern Afar, eastern parts of Benishangul Gumuz, western parts of Gambela, northern parts of SNNPR, and western parts of Oromia.

To improve the low levels and inequalities of effective coverage of newborn postnatal care in Ethiopia, national policy and programming efforts must prioritise a combined focus on accessibility and high-quality care provision, especially for disadvantaged populations. Strengthening healthcare systems at all levels is crucial, which can include healthcare worker training, health infrastructure development and improvement, provision of medical supplies and equipment, and health information system strengthening for progress monitoring and evaluation. Additionally, to improve equity in access, targeted interventions such as financial initiatives, community-based interventions, and improving the status of women are recommended. Tailored interventions based on the specific needs of each region should be implemented through close collaboration with local communities. More investment is needed for newborn postnatal care services, especially in regions with low effective coverage. Overall, multisectoral collaboration is crucial to ensure optimal, equitable, and high-quality newborn postnatal care. Future research should expand on why high-quality newborn postnatal care remains low and should integrate the multiple domains of quality of care into the crude measures for a better understanding of health gains.
